# Single-Port Transvesical Robotic Radical Prostatectomy in a Patient with Hostile Abdomen

**DOI:** 10.1590/S1677-5538.IBJU.2024.0333

**Published:** 2024-08-25

**Authors:** Sij Hemal, Sina Sobhani

**Affiliations:** 1 University of Southern California Norris Comprehensive Cancer Center Institute of Urology Los Angeles CA USA Institute of Urology, Norris Comprehensive Cancer Center, University of Southern California, Los Angeles, CA, USA

## Abstract

**Introduction:**

Robotic Radical Prostatectomy using the Da-Vinci Single-Port (SP) robot can provide comparable functional and oncological outcomes with potential advantages pertaining to peri-operative morbidity, especially in patients with an extensive history of prior abdominal surgeries (1, 2).

**Materials and Methods:**

Our case is a 74-year-old male with a history of diabetes, cardiac bypass, hypertension, and hyperlipidemia, presenting with a PSA of 7.2. His MRI showed a PIRADS-5 lesion in the left apex and mid-gland peripheral zone, and he was diagnosed with unfavorable intermediate-risk prostate cancer after MRI guided fusion biopsy. His BMI was 31, and past surgical history was pertinent for two exploratory laparotomies due to gunshot wounds and a colostomy creation followed by reversal. The standardized steps of robotic radical prostatectomy were carried out using SP robotic platform performed by author SH (3, 4).

**Results:**

Total operative time and estimated blood loss were 210 minutes and 150mL respectively. The patient was discharged on postoperative day one and final pathology showed adenocarcinoma of the prostate Gleason score 4+3=7, pT2NxR0 and negative surgical margins. The patient was continent four weeks after surgery and the PSA continues to be undetectable after three months.

**Conclusion:**

Transvesical Radical prostatectomy using the single port platform provides acceptable oncological and functional outcomes and quicker recovery given decreased risk of ileus and peritoneal irritation. Given that the abdominal cavity is not violated, the risk of bowel or vascular injury is mitigated, especially in patients with a hostile abdomen.

**Available at:** http://www.intbrazjurol.com.br/video-section/202403333_Hemal_et_al
